# Exploring the Metabolic Impact of FLASH Radiotherapy

**DOI:** 10.3390/cancers17010133

**Published:** 2025-01-03

**Authors:** Febe Geirnaert, Lisa Kerkhove, Pierre Montay-Gruel, Thierry Gevaert, Inès Dufait, Mark De Ridder

**Affiliations:** 1 Department of Radiotherapy, Universitair Ziekenhuis Brussel, Vrije Universiteit Brussel, 1090 Brussels, Belgium; febe.geirnaert@vub.be (F.G.); lisa.kerkhove@vub.be (L.K.); thierry.gevaert@uzbrussel.be (T.G.); ines.dufait@vub.be (I.D.); 2 Radiation Oncology Department, Iridium Netwerk, 2610 Antwerp, Belgium; pierre.montay-gruel@uantwerpen.be; 3 Antwerp Research in Radiation Oncology (AreRO), Center for Oncological Research (CORE), University of Antwerp, 2020 Antwerp, Belgium

**Keywords:** FLASH radiotherapy, FLASH effect, ultrahigh dose rate radiotherapy, metabolism, reactive oxygen species, mitochondria, lipid peroxidation

## Abstract

FLASH radiotherapy (FLASH RT) is an innovative modality in cancer treatment that delivers ultrahigh dose rates (UHDRs), distinguishing it from conventional radiotherapy (CRT). FLASH RT has demonstrated the potential to enhance the therapeutic window by reducing radiation-induced damage to normal tissues while maintaining tumor control, a phenomenon termed the FLASH effect. Despite promising outcomes, the precise mechanisms underlying the FLASH effect remain elusive and are a focal point of current research. This review explores the metabolic and cellular responses to FLASH RT compared to CRT, with particular focus on the differential impacts on normal and tumor tissues. Key findings suggest that FLASH RT may mitigate damage in healthy tissues via altered reactive oxygen species (ROS) dynamics, which attenuate downstream oxidative damage. Studies indicate the FLASH RT influences iron metabolism and lipid peroxidation pathways differently than CRT. Additionally, various studies indicate that FLASH RT promotes the preservation of mitochondrial integrity and function, which helps maintain apoptotic pathways in normal tissues, attenuating damage. Current knowledge of the metabolic influences following FLASH RT highlights its potential to minimize toxicity in normal tissues, while also emphasizing the need for further studies in biologically relevant, complex systems to better understand its clinical potential. By targeting distinct metabolic pathways, FLASH RT could represent a transformative advance in RT, ultimately improving the therapeutic window for cancer treatment.

## Simple Summary

FLASH radiotherapy (FLASH RT) is an emerging cancer treatment that protects healthy tissue from radiation damage while still effectively targeting superficial tumors, a phenomenon referred to as the FLASH effect. This treatment utilizes ultrahigh dose rates (UHDRs), distinguishing it from conventional RT (CRT). Despite its promising potential, the underlying mechanism explaining the FLASH effect remains undetermined, necessitating further investigations. This review discusses the current knowledge regarding the impact of FLASH RT on cellular metabolism and its limitations. Continued research is required to gain insight into the biological effects of FLASH RT and to enhance its translation to clinical settings.

## 1. Introduction

Radiation therapy (RT) is an established standard of care for cancer treatment, with more than half of cancer patients encountering RT during the course of their disease, of which 40% result in a curative treatment [1]. With cancer burden expected to affect 26 million patients by 2030, there is a pressing need to maximize the efficiency of RT [2,3,4]. The overarching goal of RT is to locally control tumors while sparing normal tissue toxicity, a concept known as the therapeutic window of RT. Nonetheless, the effect of RT remains dose-dependent, where the tumor targeting, also referred to as tumor control probability (TCP), correlates with normal tissue complication probability (NTCP). This relationship indicates that while radiation doses can increase TCP, they lead to an increase in NTCP, thus raising the risk of normal tissue toxicity. Substantial progress has been made over the years to improve the therapeutic window of RT, including the identification of new radiosensitizers and advancements in intensity-modulation and image guidance of RT. More recently, the use of ultrahigh dose rates (UHDRs) has been shown to protect normal tissue, without compromising the anti-cancer effects [5,6,7,8,9]. This biological outcome is termed the FLASH effect, characterized by delivering radiation doses at rates at least ten times faster than the conventional dose rate (DR) (0.5–5 Gy/min) [10]. With equivalent tumor control efficacy compared to conventional radiotherapy (CRT), FLASH radiotherapy (FLASH RT) has the potential to expand the therapeutic window of RT, resulting in an improved TCP/NTCP ratio, and thus, addressing the major challenge in modern RT.

Emerging research suggests that the cellular responses of normal tissue exposed to FLASH RT is different to those induced by conventional radiotherapy (CRT). While it remains elusive which response plays the overarching role, current studies focusing on the differential responses include the effect of FLASH RT on DNA damage [11,12], reactive oxygen species (ROS)-mediated oxidative stress [13,14,15], organelle targeting [16,17], cell death [18,19], oxygen depletion [20,21], and the tumor microenvironment [22,23]. Several of these topics are discussed in detail elsewhere [24,25]. An important hallmark of cancer is the plasticity of cellular metabolism [26], enabling the escape of detrimental effects of stress conditions including RT-induced cytotoxicity, making them distinct from their healthy counterparts. This metabolic plasticity contributes to a cancer cell’s inherent resistance to RT, in contrast to the radiosensitivity typically observed in normal cells [27,28].

**Figure 1:**
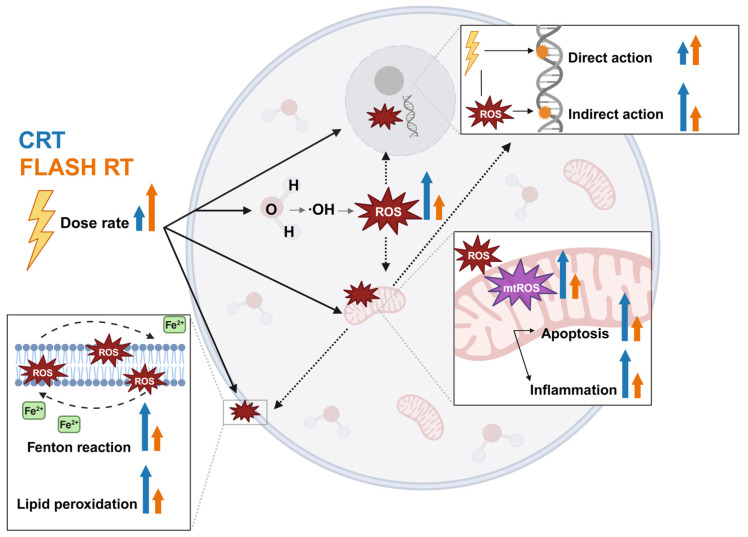
Schematic overview of cellular metabolic impact of FLASH radiotherapy (FLASH RT) (orange) compared to conventional radiotherapy (CRT) (blue). FLASH RT is delivered at an ultrahigh dose rate (UHDR), resulting in reduced reactive oxygen species (ROS) generation compared to CRT. This reduction minimized damage to normal tissues by preserving the integrity of mitochondria, lipid membranes, and nuclear DNA. Ionizing radiation (IR) generates ROS through water radiolysis, including hydroxyl radicals (∙OH). (**Top right frame**): IR damages DNA directly and indirectly via ROS-mediated damage. (**Lower right frame**): IR-induced mitochondrial ROS (mtROS) triggers apoptotic and inflammatory responses. (**Lower left frame**): IR induces oxidative stress in the lipid membrane, where labile iron (Fe^2+^) catalyzes ROS production, resulting in lipid peroxidation. This schematic highlights the distinct biological impacts of FLASH RT compared to CRT, which may play a role in the FLASH effect.

In this review, we highlight the studies that investigate how cellular metabolism rewires in response to FLASH RT and how these responses differ from those induced by CRT (Figure 1). We build on existing reviews by providing a detailed radiobiological perspective on the metabolic alterations induced by FLASH RT [29,30,31]. Where applicable, the differential behavior of normal tissue and tumors are explored. This review contextualizes the current preclinical data and theories surrounding the FLASH effect with a focus on metabolic pathways. Understanding the cellular metabolic response is crucial for optimizing the therapeutic window, predicting side effects, and facilitating the translation of FLASH RT to a clinical setting.

## 2. Redox Metabolism and FLASH RT

### 2.1. Ionizing Radiation-Induced Redox Imbalance and DNA Damage

**Figure 2:**
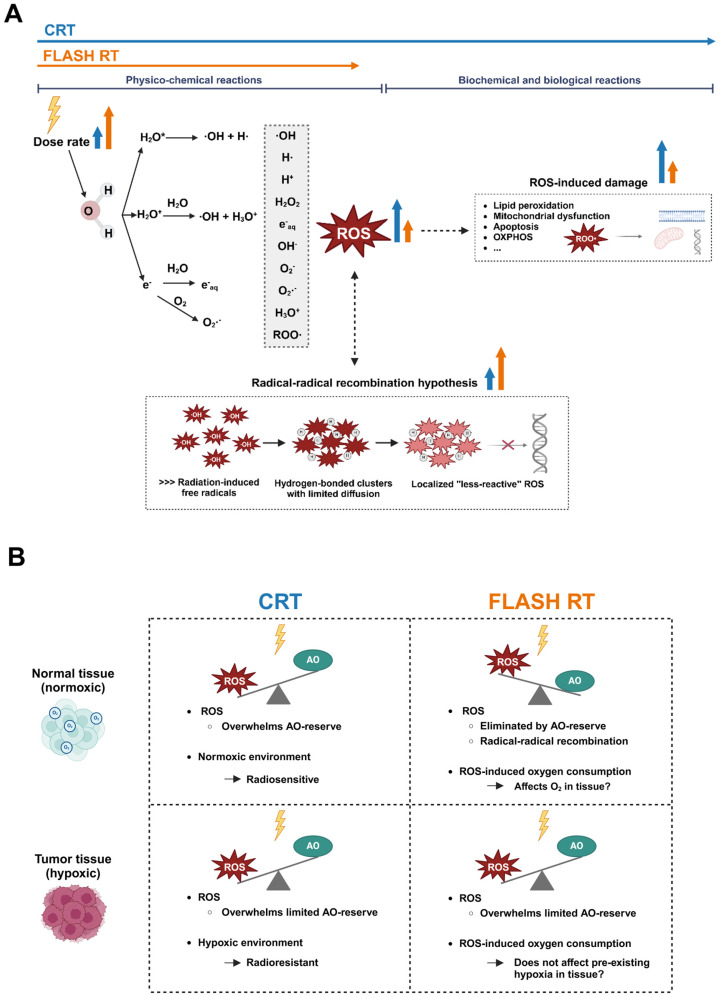
(**A**) Differential response of ROS dynamics in FLASH radiotherapy (FLASH RT) (orange) compared to conventional radiotherapy (CRT) (blue). Dose rate (DR) and ionizing radiation (IR): the left panel shows the interaction between IR and water molecules, highlighting the higher DR difference in FLASH RT, which results in more ionization events within a shorter time frame compared to CRT. Radiolysis of water: IR induces water radiolysis, producing reactive oxygen species (ROS), including hydroxyl radicals (∙OH), which form organic peroxides (ROO∙s to ROOHs). The right panel demonstrates that FLASH RT results in lower ROS levels, reducing oxidative damage to biological processes. Radical–radical recombination hypothesis: the bottom panel suggests that rapid radical formation increases recombination, creating hydrogen-bonded clusters that localize ROS and reduce DNA damage. Grey panel from top to bottom: hydroxyl radical (∙OH), hydrogen radical (H∙), hydrogen proton (H^+^), hydrogen peroxide (H_2_O_2_), aqueous electron (e^−^_aq_), hydroxide ion (OH^−^), superoxide anion (O_2_^−^), superoxide radical (O_2_∙^−^), hydroxonium (H_3_O^+^), and peroxyl radical (ROO∙). (**B**) Side-by-side comparison of ROS dynamics following FLASH RT versus CRT in normal and tumor tissue. (**Top left**): CRT increases ROS levels in normal tissue, overwhelming its antioxidant (AO) reserve, with the oxygen-rich environment rendering it particularly sensitive to CRT. (**Top right**): FLASH RT leads to lower ROS levels in normal tissue, attributable to the higher AO reserve and radical–radical recombination. FLASH RT-induced ROS also contributes to oxygen consumption, potentially affecting local oxygen levels. (**Bottom left**): CRT elevates ROS levels in tumor tissue, overwhelming its limited AO capacity. Tumor hypoxia further contributes to radioresistance. (**Bottom right**): FLASH RT generates higher ROS levels in tumor tissue, which lacks a sufficient AO pool for neutralization, while the radical–radical recombination remains relevant in this context. While ROS dynamics may influence oxygen consumption, the impact is hypothesized to be minimal in hypoxic tumor tissues.

Ionizing radiation (IR) in traditional low-linear energy transfer (LET) radiation therapy induces direct or indirect DNA damage [32]. The direct effect of IR involves the formation of organic radicals primarily on DNA, with additional impacts on other biomolecules such as proteins and lipids. The direct effect accounts for approximately a third of total radiation-induced damage [32]. The indirect effect of IR goes through the ionization of water molecules (water radiolysis) contained in biological tissues, leading to the production of reactive oxygen species (ROS) which are responsible for about two thirds of radiation-induced damage to biological molecules, including DNA [32]. These ROS include hydroxyl radicals (∙OH), superoxide (O_2_∙^−^), hydrogen peroxide (H_2_O_2_), and organic (hydro)peroxides (ROO∙s or ROOHs), as well as metal ions including labile iron (Fe^2+^), with the fixation of this damage requiring the presence of oxygen (Figure 2A). The induction of oxidative stress results in a multitude of cell responses, including signaling pathways and metabolism disruption. Agents such as arsenic trioxide [33] and cisplatin [34] exploit redox disruption to improve therapeutic responses to RT.

The mechanism underlying the capability of FLASH RT in protecting normal tissue without compromising the anti-tumor effects is yet to be elucidated, compelling researchers to test various hypotheses. A counterintuitive finding is that with CRT, lowering the dose rate (DR) encourages DNA damage repair processes, potentially reducing the risk of normal tissue toxicity [35,36]. However, FLASH RT challenges this concept by describing improved outcomes by largely increasing the DR, thereby questioning the established knowledge on redox metabolism and RT [9,37,38].

### 2.2. Conceptual Framework of ROS Dynamics in FLASH RT

Based on physico-chemical principles, including the differences in redox metabolism of cancer and normal cells, studies have proposed a theoretical framework as to why FLASH RT mitigates normal tissue toxicity [21,39]. Spitz et al. re-evaluated differences in the FLASH-induced ROS and their involvement in subsequent reactions. Calculated on assumed dose rates (0.05 Gy/s for conventional dose rate (CDR) and 40 Gy/s for FLASH DR), FLASH RT would induce approximately 36.000 to 45.000 more ionization events as opposed to CDRs [39]. It is important to note that, for a similar dose, the number of ionization events remains identical between FLASH RT and CRT, but UHDR is responsible for the production of more free radicals in a shorter time compared to CRT. Based on these theoretical ionization events, the initial hypothesis explaining the FLASH effect proposed that a FLASH pulse has the ability to consume all the oxygen present in both normal and tumor tissues [39], thus preventing the fixation of further radiation-induced damage produced by ROS. Nonetheless, this would suggest that as the NTCP decreases, the TCP would also decline. However, emerging findings have led to a more nuanced understanding of its role, refuting this hypothesis [40,41,42,43,44].

Water radiolysis products, such as ∙OH, are generated following exposure to UHDRs. An overview of these reactions and their rate constants are summarized by another study [45]. The spatially distributed ∙OH that is directly formed from water ionization following FLASH RT has the ability to, in the presence of organic molecules, undergo reactions to form highly reactive carbon-centered radicals that react with oxygen, to give rise to ROO∙. The formation of ROO∙ lays at the foundation of the radiosensitizing characteristics of oxygen following IR [46]. Its interactions with other molecules in the context of FLASH RT will be discussed in the following paragraphs.

ROO∙ is prone to undergo peroxidative chain reactions, which in DNA structures lead to bond breakage [46]. However, this is quickly followed by oxygen consumption and hydrogen abstraction, leading to the production of a ROOH population [39]. During this ROO∙ decay, superoxide and oxygen are released. The released oxygen can be removed by hydrated electrons (e^−^_aq_) and hydrogen radicals (H∙), which are generated when water is radiolysed. Therefore, this oxygen release may be viewed as a limiting factor in the oxygen depletion process following FLASH RT in tissues. Notably, ROO∙s are primarily found in lipids of the cells, which are rich in polyunsaturated fatty acids (PUFAs) [47]. Here, and in other organic molecules, the radicals participate in a lipid peroxidation chain reaction [48]. The effect of FLASH RT on lipid metabolism of the cells will be further elaborated on in another section of this review (Section 4).

The superoxide formed during the oxidation of ROO∙ can dismutate to H_2_O_2_, which would theoretically consume oxygen molecules [39]. Another portion of superoxide can activate redox-active proteins, resulting in the release of labile Fe^2+^. The ionized iron can further participate in oxidative damage or in the Fenton reaction with H_2_O_2_ [49,50]. With cancer cells containing up to four times the amount of Fe^2+^ and increased transferrin receptors compared to normal cells, which can also sequester Fe^2+^ [51,52], the Fenton reaction is suggested to contribute greatly towards the UHDR-induced ROS [39]. This further highlights the enhanced oxidative stress and associated damage observed in cancer cells compared to normal cells after FLASH RT. The effect of FLASH RT on iron metabolism will be addressed in a subsequent section of this review (Section 3).

Cancer cells are burdened by heightened levels of endogenous ROS, unlike normal cells, which have more antioxidant (AO) resources for enzymatic reduction [53]. It is suggested that the reserve of AOs in normal tissue can aid in the removal of FLASH RT-induced ROS, prior to their participation in Fenton and peroxidation chain reactions, and therefore contribute to mitigating toxicity (further discussed in Section 3 and Section 4). Consequently, considering the interactions between ROS and biomolecules, it is proposed that there is a differential accumulation of ROO∙ and ROOH in normal versus (vs.) tumor tissue [39]. However, caution should be taken when discussing the basal levels of ROS and AO systems in normal and cancer cells. The high metabolic rate of cancer cells, among other phenomena, propagates ROS. To prevent ROS from exceeding lethal thresholds, AOs are activated for ROS clearance [32,54]. The preceding statements should be interpreted with caution; while it is true that normal cells generally possess greater AO capacity, it is important to clarify that this does not imply higher absolute levels of AOs. Instead, it refers to the availability of AOs that are not pre-occupied by the task of clearing ROS.

In summary, FLASH RT affects the ROS dynamics and is assumed to alter the subsequent oxygen consumption in both normal and cancer cells during UHDR irradiation. This may occur due the spatially distributed generation of ∙OH, which gives rise to reactive ROO∙; although, this process alone does not fully explain the FLASH effect. Moreover, due to the lower levels of intrinsic ROS and more efficient elimination mechanisms during steady-state metabolism in normal cells, the reduction in ROOH is more efficient compared to tumor tissue cells, potentially illustrating how FLASH could confer protection in normal tissue without compromising on its effectiveness towards tumors (Figure 2B).

### 2.3. In Silico Insights into ROS Dynamics in FLASH RT

Having outlined the relevant radiochemical principles regarding the impact of FLASH RT on ROS, we will next discuss the in silico research and simulations addressing this topic. Simulations play a crucial role in understanding the effects of FLASH RT on ROS, particularly when examining its metabolic impact. While overviews of these effects from a physicochemical perspective are provided in previous studies [39,55,56,57], our aim in the following section is to offer a radiobiological interpretation of these simulations.

The formation of oxygen-consuming radicals upon the interaction of IR with water molecules is suggested to occur in dense clusters or spurs. A popular theory termed the radical–radical recombination hypothesis suggests that the quick formation of radicals after UHDR irradiation delivery results in a higher probability of them interacting with one another (Figure 2A) [39,58]. The increased frequency of FLASH IR leads to more interactions with water molecules, causing reactive clusters to overlap and become closer together than the initially described 100 nm separations due to dense ionization regions at the ends of electron tracks [59]. In turn, these heightened interactions would lead to a reduction in the production of ROS following FLASH RT, reducing the biological effective dose [30,57].

On the other hand, it has been argued that the diffusion-limited separation of ∙OH prevents radical–radical interactions [39], suggesting an increase in overall ROS levels with increasing DR. Various in silico studies employing Monte Carlo simulations support the idea that ROS production increases with UHDRs, through portraying the chemical evolution of ROS production following water radiolysis, primarily focusing on the detectable end-product H_2_O_2_. One hypothesis states that the ∙OH surge after UHDRs encourages recombination of this specific radical, resulting in more H_2_O_2_ [60]. Alternative hypotheses supporting elevated H_2_O_2_ levels following UHDRs assume that certain radicals rapidly recombine, resulting in increased H_2_O_2_ levels, whereas these radicals are considered as “longer-lived” following CRT [56,61,62].

One in silico study supports the radical–radical recombination hypothesis, which assumed that the immediate induction of radicals by UHDRs (or FLASH RT) increases the probability of its recombination, resulting in reduced levels of toxic ROS and thereby contributing to the mitigation of deleterious effects (in normal tissue) [20]. Based on reaction kinetics and considering the diffusion rate constant, Abolfath and colleagues demonstrated that the increased radical–radical recombination results in a decrease in certain ROS, including H_2_O_2_. Another theoretical model supporting these results portrayed that a reduction in the lifetime and concentration of toxic ROO∙ radicals likely decreases the damaging effects of FLASH RT in normal tissues [47].

The same in silico study demonstrated that the temporary hypoxic environment following FLASH RT is due to delaying hydrogen abstraction from DNA [20]. Moreover, the IR-induced radicals (including ∙OH) interact with each other forming clusters of metastable oxygen–hydrogen complexes. Consequently, instead of freely diffusing to DNA, the complexes are electrostatically kept together by hydrogen bonds, and the subsequent hydrogen abstraction from DNA is slowed down. This may suggest that the production of damaging ROS, including H_2_O_2_, may be suppressed due to the more efficient interactions of these radicals in clusters [58]. According to this hypothesis, ROS-induced DNA damage or the indirect effect of IR seems less likely to be involved in FLASH RT vs. CRT [20].

The non-homogenous effects of ROS following FLASH RT were further demonstrated mathematically [20]. In this model, the radicals generated from water radiolysis scale linearly, whereas the clusters formed after oxygen and water disintegration follow a quadratic model. This trend can be explained by the rapid conversion of, for example, ∙OH or H_2_O_2_ into the “less-reactive” ROS population of ∙OH- or H_2_O_2_- clusters. This specific ROS population of transient clusters exhibits less diffusivity, localizing them, and in turn, reducing its capacity to damage DNA. With CDRs, the ROS population remains unconverted during the period when cellular responses, that include DNA repair process and AO systems, are upregulated [20]. This provides a possible framework as to why toxicity in normal tissue is mitigated after FLASH RT (vs. CRT).

Models based on radiation chemistry principles and in silico simulations evidently have limitations. The reaction rate constants that are included are often derived from a homogenous solution, whereas their actual value can vary depending on (heterogenous) tissue type [47]. Additionally, except to a certain degree in Spitz et al.’s paper [39], these models do not consider the presence of biomolecules, such as how ROO∙ has a higher affinity for lipid substrates [57]. While previous simulations and analyses based on radiochemical concepts, excluding a singular simulation [20], predict more ROS (specifically H_2_O_2_) following UHDRs, recent studies incorporating measurements of biological parameters indicate otherwise. These studies will be discussed in the following sections (Section 2.4 and Section 2.5)

To summarize, early theories on the FLASH effect focused on the production of oxygen-centered radicals (e.g., ∙OH) and carbon-centered radicals (which can form ROO∙), which were believed to consume oxygen through downstream reactions [21,39]. However, recent findings demonstrate that the magnitude of oxygen depletion alone is inconsistent with the biological effects observed following FLASH RT, though it likely influences the preservation of normal tissue [40,41,42,44]. Additionally, the surplus of AOs in normal tissue cells may counteract ROS-induced damage, while tumor cells remain susceptible. This selective sparing may also be attributed to heightened radical–radical interactions, limiting ROS diffusion to cellular components, including DNA. Yet, the predominant reaction driving the FLASH effect is still to be determined.

### 2.4. In Vitro Insights into ROS Dynamics in FLASH RT

Montay-Gruel et al. demonstrated long-term neurocognitive sparing in tumor-free mice models following electron FLASH RT (>100 Gy/s) compared to CRT (0.07–0.1 Gy/s) [13]. To explore the role of ROS in the FLASH effect, H_2_O_2_ levels were quantified in cell-free water solutions using a radiochemical assay with an H_2_O_2_ probe. The oxygen tension was equilibrated at 4% which represents the physiological concentration in the brain of approximately 30 mmHg [63]. In accordance with the in silico simulation [20], lower levels of H_2_O_2_ were detected in pure water after FLASH RT, compared to CRT [13]. These results were substantiated by another study demonstrating lower H_2_O_2_ yield after UHDR radiation in water radiolysis experiments, regardless of the temporal structure of the beam (electron (≥1400 Gy/s) vs. proton beams (0.1 and 1260 Gy/s)) [64]. In other words, H_2_O_2_ concentrations increased proportionally with the dose and were inversely proportional with DR, in pure water. Others additionally observed that as the average electron DR increased (0.14 to 1500 Gy/s), both H_2_O_2_ and oxygen levels decreased: with an estimated 7.51 moles of H_2_O_2_ produced for every mole of oxygen consumed [65]. Furthermore, research validated lower H_2_O_2_ production in pure water following proton UHDR (40 Gy/s to 60 kGy/s) and subsequently suggested the previously described radical–radical recombination theory to lay at the base of this phenomenon [58]. At UHDRs, overlapping ROS spurs increase the interactions of neighboring radicals, which suppress the H_2_O_2_ production. While water radiolysis products are formed within microseconds, oxygen depletion and other more complex biochemical processes (including AO upregulation and DNA repair) are considered slower steps [58]. Although not demonstrated with H_2_O_2_, others revealed a similar relationship between increasing protons DR and a reduction in the yield of 7-hydroxy-coumarin-3-carboxylic acid (7OH-C3CA). Specifically, a solution with coumarin-3-carboxylic acid (C3CA), which acts as a ROS scavenger, captures ∙OH in the presence of carbon ions and protons, leading to the formation of 7OH-C3CA. Moreover, they found that the reduction rate was higher than oxygen depletion with UHDRs, suggesting that it is indeed the radical–radical interactions that could be highly involved in the FLASH effect [66]. Nonetheless, older studies have reported the opposite, specifically, that UHDRs produce more H_2_O_2_ in aqueous solution compared to CDRs [67,68,69].

A recent study further investigated the effect of FLASH on water radiolysis, by including CO_2_ in aqueous solutions and comparing high-LET (carbon ions at 50 and 0.1 Gy/s) and low-LET electrons (600 and 0.62 Gy/s) and x-rays (10 and 0.1 Gy/s) [45]. They found that, regardless of the type of radiation (high or low LET) and the presence of oxygen or carbon dioxide (CO_2_), UHDRs consistently reduced H_2_O_2_ levels compared to CDRs. CO_2_ was included as it can increase H_2_O_2_ production through lowering pH and is an important molecule in all physiological environments. With the use of e^−^_aq_ scavengers (N_2_O and NaNO_3_), the study demonstrated that e^−^_aq_ can eliminate ∙OH, the precursor of H_2_O_2_. This illustrated how e^−^_aq_ is responsible for the decreased levels of H_2_O_2_ after UHDRs in pure water.

This study further explored the Monte Carlo simulation theory that describes that upon CRT, long-lived radicals from previous pulses would affect the dynamics of newly formed ROS, resulting in reduced H_2_O_2_ production [56,61,62]. In UHDRs, the three-pulsed UHDRs showed a lower H_2_O_2_ yield compared to the one-pulsed UHDR, for low-LET IR [45]. However, this was not observed with high-LET IR, likely due to variety in radical distribution and oxygen production between the two LET conditions. A similar study investigated the differential effect of a high- (proton beam (80 Gy/s)) and low-LET (electron beam (660 Gy/s)) with UHDRs on ROS yield [70]. Interestingly, the yield of ∙OH was not dependent on beam type, while the more stable H_2_O_2_ did show a significant reduction following IR with electrons vs. protons, in line with the study by Zhang and colleagues [45].

More recently and biologically relevant, radiation-induced cellular ROS were quantified in a model of human fibroblasts (IMR90), irradiated with 15 Gy protons at a CDR (0.33 Gy/s) or with UHDRs (100 Gy/s) [19]. While CRT steadily increased ROS levels, measured with a 2′,7′-dichlorofluorescin diacetate (DCFDA) probe, no changes occurred after exposure to FLASH RT, confirming the data gathered in the studies described above in cell-free cultures.

In summary, FLASH RT results in lower H_2_O_2_ production compared to CRT, which is attributed to radical–radical interactions at UHDRs, and this is further supported by studies showing no ROS increase in human fibroblasts following FLASH RT.

### 2.5. In Vivo Insights into ROS Dynamics in FLASH RT

In the context of neuroprotection, mice were subjected to carbogen breathing (95% O_2_/5% CO_2_) before and during electron FLASH RT (>100 Gy/s) with the aim to increase radiation-induced indirect damage [13]. Here, the initial neuroprotection in the brain provided by FLASH RT (compared to CRT (0.07–0.1 Gy/s)) was significantly diminished, indicating the importance of ROS and local oxygen concentration to obtain the FLASH effect. In the same study, zebrafish embryos were irradiated with FLASH RT or CRT in the presence of AOs: amifostine and N-acetyl cysteine (NAC) [13]. While CDRs induced more detrimental effects compared to FLASH RT on zebrafish embryos, as determined by changes in body length, both ROS scavengers had minor protective effects on the zebrafish embryos after UHDRs. This further suggests that ROS is less likely to contribute to the therapeutic effect of FLASH RT, while playing an established role in CRT. Others similarly observed full recovery of zebrafish embryos after UHDRs and calculated that morphology preservation was correlated to a production of 2.33 molecules of H_2_O_2_/100 eV or less [64]. While this was not dependent on beam and dose rate when using protons (0.1 and 1260 Gy/s), it was the case for electron irradiation (≥1400 Gy/s). Whether this value could be considered a threshold for the FLASH effect remains undetermined [64,71].

Another study measuring ROS in subcutaneous murine lung carcinoma (LLC) tumors using a DCFDA probe demonstrated increased levels of ROS following 15 Gy FLASH RT (40 Gy/s) compared to CRT (0.01 Gy/s) [72]. However, it is noteworthy that the increased intracellular ROS were not paired with a subsequent increase in DNA double-strand breaks (DSBs). Similarly with these findings, an elevation in ROS levels in the small intestines of electron FLASH-irradiated (>150 Gy/s) mice with 15 Gy was observed, as demonstrated by dihydroethidium (DHE) staining [73]. Additionally, the levels of superoxide dismutase (SOD), used as surrogate marker for AO activity, were comparable between the FLASH RT and CRT groups. However, MDA concentration, an indicator for lipid peroxidation, was significantly lower in the FLASH-irradiated mice (vs. CRT). Despite increased ROS, FLASH RT appears to have better control over lipid damage, suggesting that the downstream effects of ROS contribute to the protective effect observed with FLASH RT, while ROS scavengers, like SOD, play a lesser role [73]. Nonetheless, the effect of FLASH RT on lipid peroxidation will be further explored in the lipid metabolism section of this review. This was in contradiction to Montay-Gruel’s study, which showed reduced H_2_O_2_ levels after UHDRs [13].

To recapitulate, FLASH RT’s neuroprotective effects are compromised by carbogen breathing, highlighting the role of ROS and oxygen levels in the FLASH effect. Meanwhile studies in zebrafish and mice reveal that, despite higher ROS levels, FLASH RT offers improved management of lipid damage and does not rely on AO for its therapeutic benefits.

### 2.6. Challenges in Measuring ROS Dynamics in FLASH RT

Given the discrepancy in the concentrations of ∙OH vs. H_2_O_2_ necessary to induce equivalent levels of toxicity [74,75], this raises the question whether H_2_O_2_ is the appropriate species for quantifying ROS yield after IR or if overall ROS would be more beneficial. Specifically, Koch et al. highlighted that to induce the same level of toxicity as the ∙OH generated by 10 Gy, a 50.000-fold higher concentration of H_2_O_2_ would be needed. Additionally, the initial generation of H_2_O_2_ following FLASH IR is likely short-lived, as it is quickly scavenged by peroxidases [56]. Measuring overall ROS with a DCFDA-probe or via DHE staining has provided a different perspective on ROS production following FLASH RT [72,73], rather than quantifying H_2_O_2_ as a ROS surrogate [13]. Nonetheless, the study on human fibroblasts showed consistent reductions in DCFDA [19], consistent with the decrease observed for H_2_O_2_ in aqueous solutions [13]. The observed increases in ROS likely reflect the immediate abundance of ionization events caused by FLASH RT, while observations in H_2_O_2_ levels may result from “slower” secondary reactions derived from primary radical species. This raises further questions about whether each type of ROS can be considered equally important and whether these contradictory changes in ROS levels reported in the studies are truly biologically significant.

The inconsistency in ROS results may be attributed to the vast differences between their study in cell-free systems and heterogenous tumor environments. G-values, which convert the IR dose to a radical concentration and depict ROS yield, are greatly dependent on the density of organic molecules. For instance, per loss of oxygen, radiolysis of culture medium yields a G-value of −0.44 µM/Gy [76,77] and further rises to −0.68 µM/Gy when cells are added [39,78]. This is especially relevant when applying these results to the effects observed after FLASH RT in the brain, which is rich in PUFAs that can significantly enhance the G-value [75,79]. To account for the lack of organic material, some studies add albumin to the water to mimic in vitro and in vivo protein content, which also serves as a substrate for ROS reactions, partially addressing this issue [65,70]. This underscores the impact of organic density on ROS yield, a component lacking in studies performed in pure water, presenting a major limitation.

On the flip side, in the field of water radiolysis studies, cell culture systems have inherent disadvantages. The water-to-cell volume ratio is much larger in cell cultures compared to tissues, meaning that there is more oxygen per cell available in the cell cultures [39]. This difference supports the proposal that oxygen depletion associated with the FLASH effect is non-existent in cell lines cultured at 21% oxygen, due to the abnormally high oxygen tension [29]. Vozenin et al. suggested that doses up to 90 Gy are necessary to make these cell cultures anoxic [29], further arguing against the use of cell culture systems for investigating ROS. Indeed, the hypoxic conditions in the pure water solutions were 1% and 4% O_2_ (in addition to normoxic 21% O_2_ water) [13,45], representing tumor hypoxia and normal tissue tension or pO_2_ in the brain, respectively. Nonetheless, we cannot overlook the fact that there are experimental setups, including hypoxia incubators and hypoxia chambers, that allow for the culture and irradiation of cell lines under controlled oxygen conditions.

Overall, preliminary research is mostly limited to studies performed in aqueous solutions, which are distant from investigations involving cell lines or human-relevant models, stressing the gap between these findings and their applicability to a biological context.

## 3. Iron Metabolism in Ionizing Irradiation

As previously stated, the biological effects of IR can be primarily attributed to the formation of water radiolysis products such as ∙OH and H_2_O_2_, but labile iron (Fe^2+^) and lipid metabolic enzymes also play important roles [80,81]. Following (conventional) IR, superoxide is generated and can be converted to H_2_O_2_ via SOD. H_2_O_2_ may then participate in the Fenton reaction, where it reacts with the labile iron pool to produce hyperactive ∙OH radicals [82]. These radicals are known to generate DNA DSBs, but preferably interact with PUFAs, which can give rise to lipid hydroperoxides, and subsequent oxidative damage [83].

The accumulation of lipid peroxidation is linked to a relatively new form of cell death: ferroptosis. This form of iron-dependent programmed cell death has been observed in irradiated tumors [84]. Additionally, evidence reported a link between ferroptosis and fibrosis, a well-known late side effect of RT in normal tissue [85]. Cancer cells, characterized by abnormal iron homeostasis, have increased iron demands due to their heightened metabolism and proliferation. Iron transport relies on circulating transferrin and iron storage proteins like ferritin, while ferroportin acts as an iron exporter [83,86]. The increased iron pool leads to more oxidative stress, but cancer cells counteract this via upregulating their AO defenses [39]. Notwithstanding, iron is an important factor for cell proliferation and thus, cancer cells are more prone to iron depletion compared to normal cells [86]. Cancer cells that are therapy-resistant or metastatic are more prone to ferroptosis [87,88,89].

While the effects of IR on iron and lipid metabolism are intertwined, we will address the effects of FLASH RT on these processes separately to maintain clarity in this review.

### 3.1. Conceptual Framework of Iron Metabolism in FLASH RT

In addition to the differences between normal cells and tumor cells in their ability to eliminate FLASH-induced ROS (ROO∙ and ROOH), the content of redox active iron, i.e., labile iron (Fe^2+^), might also be involved in the differing effects of FLASH RT between normal and tumor tissues. This provides a hypothesis to explain the enhanced therapeutic window observed with FLASH RT.

**Figure 3:**
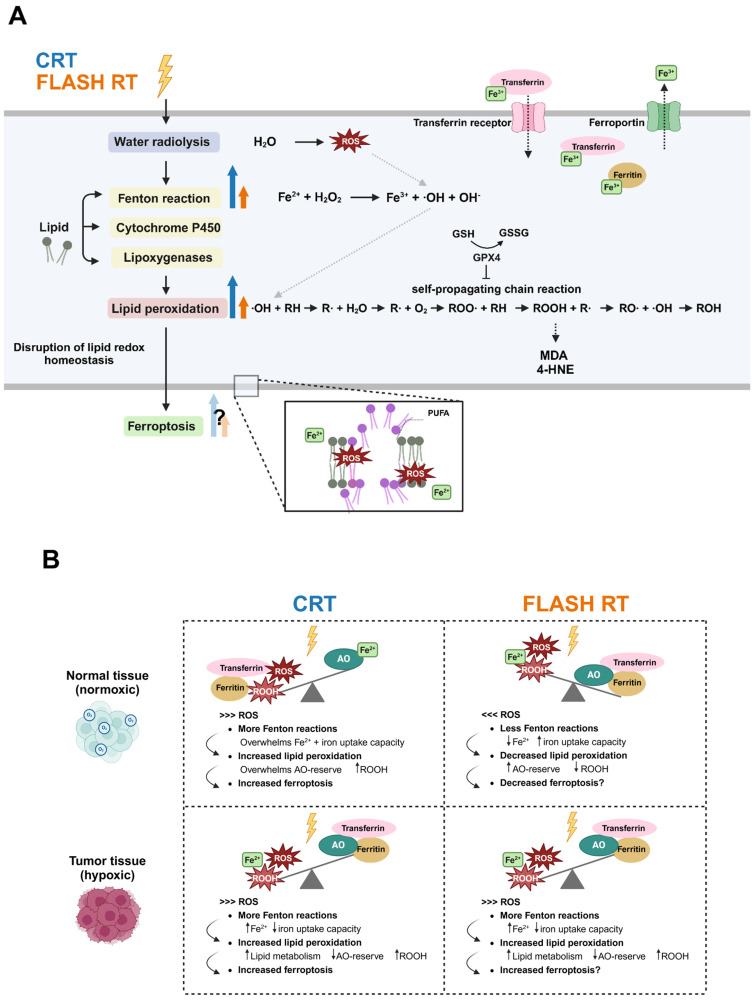
(**A**) Differential impact on iron and lipid metabolism following FLASH radiotherapy (FLASH RT) (orange) compared to conventional radiotherapy (CRT) (blue). Reactive oxygen species (ROS) generated from water radiolysis interact with iron-containing proteins leading to Fe^2+^ release. Iron transport relies on circulating transferrin, ferritin which stores iron, and ferroportin acting as an iron exporter. Labile Fe^2+^ can participate in Fenton reactions with hydrogen peroxide (H_2_O_2_) and organic or lipid hydroperoxides (ROOHs) generating hydroxyl radicals (∙OH) that further fuel ROS production, increasing oxidative stress. ROS can oxidize biomolecules, including polyunsaturated fatty acids (PUFAs) in phospholipids, initiating lipid peroxidation. Lipid peroxidation is a chain reaction that can be initiated via non-enzymatic Fenton reactions and enzymatic pathways with cytochrome P450 and lipoxygenases. Lipid hydroperoxide (ROOH) accumulation result in a self-propagating reaction that can trigger ferroptosis, a form of iron-dependent cell death. Additionally, lipid hydroperoxides (ROOHs) give rise to malondialdehyde (MDA) and 4-hydroxynonenal (4-HNE). Antioxidants (AOs), including glutathione (GSH) and glutathione peroxidase 4 (GPX4) inhibit this cascade. (**B**) Side-by-side comparison of iron and lipid metabolism after FLASH RT versus CRT in normal and tumor tissue. (**Top left**): CRT elevates ROS levels in normal tissue, increasing Fenton reactions despite high iron sequestration capacity. Oxidative stress is further enhanced by increased lipid peroxidation, overwhelming antioxidant (AO) reserves. This results in the accumulation of lipid hydroperoxides (ROOHs), which can trigger ferroptosis, contributing to normal tissue toxicity. (**Top right**): reduced ROS levels after FLASH RT decrease the activation of Fenton reactions in normal tissue. This effect is further supported by the intrinsically low levels of labile Fe^2+^ and the tissue’s efficient iron sequestration capacity. As a result, there is less lipid peroxidation due to reduced Fenton reactions and the effective elimination of ROOH by the AO reserve. Thus, the likelihood of ferroptosis occurring is hypothesized to be reduced. (**Bottom left**): CRT elevates ROS levels in tumor tissue, leading to more Fenton reactions. This effect is amplified by the higher levels of labile Fe^2+^ iron and reduced capacity for iron uptake in tumors. In turn, lipid peroxidation rises, driven by the upregulated lipid metabolism in tumors that enables them to cope with cellular stresses. The resulting accumulation of ROOH cannot be effectively eliminated by the limited AO reserve, leading to increased ferroptosis. This form of programmed cell death can be inhibited by hypoxia, which may contribute to the radioresistance of the tumor. (**Bottom right**): while FLASH RT results in less ROS formation, it is the heightened levels of labile Fe^2+^ in tumor tissue and its reduced iron sequestration capacity that fuel the Fenton reactions. In turn, lipid peroxidation is elevated, also driven by the active lipid metabolism is tumor tissue. The accumulation of ROOH overwhelms the already-limited AO reserve, potentially increasing ferroptosis. This suggests a localized effect of FLASH RT on cancer cells.

The effect of FLASH RT on iron metabolism can be seen as a sequel of reactions (Figure 3A). This has been described in a study that considered the theoretical yield of radiolytic products in soft tissue [39]. After UHDR radiation, the starting point of the chain reaction can be pinpointed to the formation of superoxide, which occurs when oxygen interacts with e^−^_aq_ and H∙ after FLASH RT [39]. Some superoxides can dismutate to H_2_O_2_ via SOD or spontaneously [39,47], while other superoxide interacts with iron-containing proteins, including ferritin, leading to Fe^2+^ release. This FLASH-induced interaction can eventually double or triple the Fe^2+^ pool due to a feedback loop [39]. In this context, labile Fe^2+^ can form complexes with oxygen or participate in Fenton reactions with H_2_O_2_ and ROOH, thereby fueling additional Fenton reactions that produce radicals like ∙OH [49,50]. In cancer cells, which typically have higher levels of Fe^2+^ and more transferrin receptors [51], the enhanced radical production makes them particularly vulnerable to FLASH RT (Figure 2B). In contrast, normal tissue cells contain less Fe^2+^ and fewer transferrin receptors, along with an increased capacity to effectively sequester Fe^2+^ due to their higher AO reserve [51,52]. This allows them to limit the availability of Fe^2+^ for Fenton reactions and more effectively manage the surge of ROS produced by FLASH RT [39]. This context offers a hypothesis as to why normal cells are protected by FLASH RT, while its antitumor effects remain effective.

Thus, the differing effects of FLASH RT on normal and tumor tissues may be explained by their ability to handle IR-induced ROS and the content of labile Fe^2+^. Cancer cells, with higher levels of Fe^2+^ and transferrin receptors, enhance radical production, making them more susceptible to FLASH RT. In contrast, normal cells have lower Fe^2+^ and robust AO reserves, enabling them to sequester iron and manage ROS more effectively, which may contribute to selective protection of normal tissue.

### 3.2. Studies into Iron Metabolism in the Context of FLASH RT

While the differential role of iron in normal and cancerous tissues has been theoretically explored through the radiochemical theories described above [39,47], studies on the impact of FLASH RT on iron metabolism using research models are scarce. To our knowledge, no in silico, in vitro, or in vivo studies have been performed to validate whether the higher levels of labile iron and transferrin in tumors, including the near-saturated system to eliminate ROS, contributes to FLASH RT’s selective targeting of tumors while protecting normal tissues which have opposite iron-related characteristics.

However, certain studies are relevant to this subject. In particular, the optimization of the Frick gel dosimeter using chelator eriochrome cyanine R (FGECR) is relevant, which has been described for accurate radiation measurement and relies on the interaction between IR and iron ions [90]. As previously discussed, the Fenton reaction converts Fe^2+^ to its oxidized form, Fe^3+^, producing highly reactive radicals and additional oxidative stress leading to cellular damage. The FGECR dosimeter measures the radiolytic conversion to Fe^3+^ via forming complexes with the ion using ECR as a chelating agent. The study found that the formation of the Fe^3+^–chelator complex, and the corresponding absorbance, increased consistently with radiation dose [90]. This could be beneficial for use in FLASH RT, as the dosimeter demonstrated a linear dose response up to high doses (40 Gy) and maintained a distinct ratio between maximum and minimum detectable doses, which is essential for measuring UHDRs in FLASH RT [91].

The role of iron in FLASH-induced oxidative damage is prominent, offering a local effect in tumors while sparing normal tissues, albeit not all normal tissue is fully protected [9]. A potential approach to enhance tissue protection could be through minimizing oxidative damage by using compounds such as cystamine, known for its AO and radioprotective properties. Researchers used a Fricke dosimeter to measure the oxidation of Fe^2+^ to Fe^3+^ as an indicator of radiation induced damage, employing Monte Carlo simulations to replicate UHDR conditions similar to FLASH RT [92]. In this in silico study, the protective impact of cystamine was evaluated. The results showed that cystamine reduced the conversion of Fe^2+^ to Fe^3+^, indicated by reduced Fe^3+^ levels [37,43]. Cystamine was found to capture IR-induced reactive species, protecting the competing Fe^2+^ from oxidation, thereby reducing the oxidative damage. This study demonstrated that cystamine provided greater protection at UHDRs (instantaneous pulses of 300- MeV) compared to CDRs. Under hypoxic conditions, the reduction of Fe^3+^ was less pronounced due to fewer radicals present to interact with Fe^2+^, reflecting the partial independence of oxygen availability [92]. Combining cystamine with FLASH RT could modulate iron metabolism, offering more radioprotection to normal tissue while potentially enhancing the therapeutic window by selectively targeting cancer cells, which are more susceptible to iron fluctuations [86].

Although studies on the impact of FLASH RT on iron metabolism are limited, existing research suggests that the differing iron levels in normal vs. cancerous tissues, along with compounds like cystamine, may enhance tumor targeting while sparing normal tissue from FLASH RT-induced oxidative damage.

## 4. Lipid Metabolism in Ionizing Irradiation

Upon interaction with water molecules in the cells, IR can generate ROS, which can oxidize biomolecules, including lipids (Figure 3A). The disruption of lipid redox homeostasis, defined as a balance between oxidized and reduced lipids, can trigger ferroptosis, a lethal form of programmed cell death. As previously stated, PUFAs in phospholipids are key substrates for lipid peroxidation [79,83], and this process can be initiated through the enzymatic pathway with lipoxygenase or cytochrome P450, or via the non-enzymatic Fenton reaction. The accumulation of lipid (hydro)peroxides results in a self-sustaining chain reaction that results in membrane rupture and eventual cell death [79,84].

Cancer cells are characterized by elevated lipid levels to meet increased energy demands for maintaining their high proliferative status [93]. The lipids can either be exogenously sourced or sourced via de novo synthesis, which is considered the most prominent pathway for lipid recruitment [94]. According to some studies, cancer cells have less cholesterol in their lipid bilayer compared to normal cells [95,96], making them more susceptible to lipid peroxidation via membrane pore-formation; although, this is dependent on cancer type and physiological conditions [97,98]. Increasing evidence has demonstrated a connection between RT, lipid redox homeostasis, and ferroptosis. Studies have demonstrated that RT disrupts lipid redox homeostasis through suppressing the antiporter that mediates uptake of cystine in exchange for glutamate, which resulted in excessive lipid oxidation and, thus, ferroptosis [84]. Additionally, RT has been shown to increase levels of ACSL4, the enzyme responsible for PUFA synthesis [99].

While both saturated and unsaturated fatty acids exist, it is the unsaturated lipids that are more sensitive to oxidative stress from RT [79]. This accumulation can be inhibited via AOs, including glutathione peroxidase 4 (GPX4) and glutathione (GSH), which reduce the lipid hydroperoxides, countering the lethal effects related to ferroptosis (Figure 3A) [100]. Moreover, ferroptosis inducers have been found to enhance the efficacy of RT [101], while ferroptosis inhibition has been recognized as a therapeutic opportunity to limit RT-induced toxicity [102]. Lastly, lipid oxidation in tumors has been linked to better treatment outcomes of patients [99].

### 4.1. Conceptual Framework of Lipid Metabolism in FLASH RT

The biological effect of IR-induced cellular damage is largely caused by ROS generation, which not only targets DNA but also other biomolecules including proteins and lipids. We previously described how FLASH RT changes ROS dynamics, which, in turn, may decrease the time frame for lipid peroxidation to propagate, thereby reducing oxidative stress in the cell membranes. While this event applies to both normal and tumor tissue, the different biological responses observed following FLASH RT lie in variations in their defense systems and Fe^2+^ pools (Figure 3B).

Evidence has shown that low DRs enhance lipid peroxidation more than high DRs [103]. Over thirty years ago, a review discussed how low DRs were associated with higher damage of lipid membranes [14]. This so called “inverse dose rate effect” was attributed to the radical–radical recombination hypothesis, which proposes that following UHDRs, there are fewer highly reactive radicals, or their lifetime is shortened, making them less available to participate in the lipid peroxidation chain reaction [14,104,105]. Thus, the hypothesis presumes that UHDRs reduce the diffusion of radicals via increased radical–radical interactions, resulting in limited ROS exposure and a shorter time frame for lipid peroxidation to propagate.

Following FLASH RT, ∙OH generates ROO∙, which is proposed to interact with biomolecules in a way that differs kinetically from CRT [39]. ROO∙, primarily localized in lipids, initiates peroxidation through abstracting hydrogens from PUFAs, yielding new radicals that have the ability to perpetuate the chain reaction (Figure 3A). In the case of lipid peroxidation, the most abundant product is lipid hydroperoxides. This process consumes oxygen and forms new ROO∙, increasing the peroxidation-induced damage to the cells [47]. Meanwhile, the lipid hydroperoxides give rise to aldehydes, including malondialdehyde (MDA) and 4-hydroxynonenal (4-HNE), which are often quantified to measure lipid peroxidation in biological experiments. The autocatalytic reaction continues until it is terminated by radical–radical annihilation, via thiol-containing compounds with reducing capacities or vitamin E [47,103,106,107]. The brain, of which 30% of the fatty acids are proposed to be PUFA [108], is particularly susceptible to lipid peroxidation, highlighting how the oxidative cascade is highly influenced by tissue type [39].

With regards to the differential biological response between normal and cancer tissue after FLASH RT, this can be attributed to certain factors (Figure 3B). First, the defense systems in normal tissues are more equipped to facilitate the removal of hydroperoxides, inhibiting their involvement in peroxidation chain reactions [39]. Furthermore, once the lipid chain reactions are initiated and propagated, normal cells, with their greater AO capacity, can terminate these reactions and limit the detrimental effects of lipid (hydro)peroxides. In contrast, cancer cells, which have an elevated lipid metabolism to cope with their high proliferative status, are more prone to lipid peroxidation [87,88,89]. This ties into the second factor potentially explaining the FLASH effect: cancer cells typically have double to quadruple the amount of labile Fe^2+^ and more iron uptake capacity (transferrin receptors), compared to normal cells [51,52]. The increased levels of Fe^2+^ catalyze the Fenton reaction, leading to more radical production in cancer cells, as described in the previous section on iron metabolism (Section 3.1). These ROS promote lipid peroxidation, of which the accumulation of oxidized lipids triggers ferroptosis. Thus, higher Fe^2+^ makes cancer cells particularly vulnerable to ferroptosis. In summary, the differences in AO homeostasis and the Fe^2+^ pool between cancerous vs. normal tissue explain the promising role of lipid peroxidation in the FLASH effect (Figure 3B).

In summary, FLASH RT impacts normal and tumor tissues differently in terms of lipid peroxidation. Normal tissues, with higher AO reserves, mitigate lipid peroxidation and oxidative stress, while tumor cells, with higher Fe^2+^ levels, are more susceptible to radical production and lipid stress. This explains the effectiveness of FLASH RT in targeting tumor cells while limiting normal tissue toxicity.

### 4.2. In Vitro Studies into Lipid Metabolism in the Context of FLASH RT

The potential involvement of lipid peroxidation in the FLASH effect, as well as its role in the oxygen depletion theory related to FLASH RT, has been explored in the following studies.

In the context of emerging FLASH RT, Froidevaux et al. explored the differential effect of 40 Gy electron CRT (0.14 Gy/s) and FLASH RT (>540 Gy/s) on lipid peroxidation [15]. The study utilized linoleic acid (LA) micelles and phophatidylcholine (PC) liposomes, with the latter representing PUFAs, as a cell membrane proxy. Differences in lipid peroxidation were quantified by measuring lipid hydroperoxides and MDA, which is a product of oxidative breakdown of lipid hydroperoxides. CRT resulted in a linear dose-dependent increase in lipid peroxidation, which was approximately halved when the oxygen concentration decreased from 21% to 4%. On the other hand, independent of oxygen level, FLASH RT did not result in lipid peroxidation. It was hypothesized that lower lipid peroxidation following FLASH RT could be due to radicals recombining before diffusing to the lipid targets, leading to less oxidative damage in the lipid bilayer, despite measuring near identical yields of ∙OH following CRT vs. FLASH RT [15].

The effect of DRs in lipid peroxidation revealed that upon exceeding 0.2 Gy per pulse, the occurrence of lipid peroxidation decreased [15], which is in line with the established observation that lower DRs result in more lipid peroxidation [105]. While the values differ, the overall trend is similar to Montay-Gruel et al.’s findings [13], where neuroprotection in mice models occurred when the DR was higher than 1 Gy per pulse [38], suggesting that the higher DR indeed results in less damaging reactions. While acknowledging that comparing chemical micelles and liposomes to an in vivo model is challenging due to their fundamentally different properties, the different dose per pulse value in vivo suggests that the FLASH effect is influenced by more than solely reduction in lipid peroxidation.

To investigate if pulse frequency and oxygen availability played a role in the reduced lipid peroxidation levels following FLASH RT, the same research group performed a follow up study that included different concentrations of liposomes, as well as varying content of LA present in the liposomes [103]. Regardless of liposome concentration or composition, FLASH RT (564.6 Gy/s) consistently led to lower lipid peroxidation and oxygen consumption compared to CRT (0.2 Gy/s). Nonetheless, MDA yield was inversely proportional to LA content in liposomes; more LA (which represents the biologically relevant PUFAs) led to less MDA. Conversely, more LA content in liposomes resulted in more oxygen consumption and lipid hydroperoxide. The following hypothesis was proposed to explain the differential effect of MDA production vs. oxygen consumption and hydroperoxides [103]: in membranes with less oxidizable lipids, the greater distance between them may favor chain termination. Due to inhibiting the propagation of the chain reaction, this would lead to higher MDA release but lower consumption of oxygen. On the other hand, membranes with closely spaced PUFAs favor chain propagation, leading to less MDA yield but increased oxygen consumption and lipid hydroperoxide. Given the prominent role of oxygen consumption in the propagation of the chain reaction, these results suggest that lipid peroxidation ameliorates in membranes with more PUFAs following FLASH RT, even though MDA levels were lower. With lipid hydroperoxides’ formation requiring oxygen, this trend in oxygen consumption appears reasonable. This study suggests an important reconsideration: instead of MDA as an indicator of lipid peroxidation, studies should focus more on oxygen consumption and lipid hydroperoxide, which seem to better reflect the propagation of the chain reaction.

Next, the study demonstrated that the average DR (the DR calculated by averaging the total dose contained in all delivered pulses over the entire time of irradiation) was a good predictor for the degree of lipid peroxidation as it showed stable correlation between MDA yield and oxygen consumption (when compared to the “instantaneous” DR, calculated for a single electron pulse) [103]. This result aligns with research previously described in the ROS section of this review, which found a correlation between the average DR and ROS production [65]. Similarly to the MDA profile, both H_2_O_2_ and oxygen consumption in aqueous solutions decreased as the average dose rate increased.

Additionally, the same study evaluated the influence of pulse frequency on lipid peroxidation [103]. As pulse frequencies increased, elevated levels of MDA and lipid hydroperoxides were observed in PC liposomes. This underscores that lipid peroxidation is influenced by not just the mean DR, but other beam parameters as well.

While LA represents the most dominant PUFA in cell membranes, the simplistic chemical model challenges the crossover to biologically relevant models. To address this issue to a certain extent, the oxylipin pool, which comprises lipid metabolites derived from the oxidation of PUFAs, in normal epithelial (RPE1-hTERT) and melanoma cancer (SK-MEL-28) cells following 20 Gy electron FLASH RT was recently investigated [109]. Of note, oxylipin can be derived from lipid peroxidation but is also the result of strictly regulated enzymatic pathways. Oxylipin levels were found to be reduced by 20–50% in normal cells after FLASH RT compared to CRT, similarly to the two previous studies demonstrating no lipid peroxidation or a lower yield of lipid peroxidation (vs. CRT), respectively [15,103]. Interestingly, this reduction in oxylipins was less pronounced under hypoxia, whereas Froidevaux et al. found no difference in MDA and lipid hydroperoxide production in their liposomes and micelles at either 21% or 4% O_2_ [15]. However, it should be recapitulated that 4% O_2_ is reflective of the concentration typical of normoxic tissue and is not classified as hypoxia. Nonetheless, these trends were not observed in tumor cells, indicating for the first time that reduction in oxylipins following FLASH RT may be unique to normal cells.

While research on FLASH RT’s impact on lipid metabolism is actively ongoing, studies suggest that FLASH RT leads to less lipid peroxidation compared to CRT, potentially due to radical recombination prior to reaching lipid targets, causing different responses in tumor vs. normal tissues.

### 4.3. In Vivo Studies into Lipid Metabolism in the Context of FLASH RT

Currently, two studies have been reported regarding the effect of FLASH RT on lipid peroxidation in vivo [109,110]. In one study, the differential remodeling of the oxylipin status in mouse lungs following 20 Gy FLASH RT was demonstrated. A downregulation of lipid derivatives was observed in the mouse lung immediately after FLASH RT, similar to the findings observed in liposomes and micelles (see previous paragraph) [15], albeit the levels had recovered after 24 h. Further alterations in oxylipins did occur in the following weeks (up to 2 months post-irradiation). The rapid reduction in oxylipins following FLASH RT is hypothesized to result from radical recombination prior to the reactions of lipid peroxidation, highlighting the significant role of cell membrane composition. This resonates with the previous observations, which emphasize the importance of the distance between PUFAs in the cell membrane [103].

Another study employed Fourier Transform Infrared Microspectroscopy (FTIRM) and Principal Component Analysis (PCA) in an attempt to map out the molecular alterations in biomolecules, including lipids, caused by electron FLASH RT [110]. The findings revealed differences in protein signatures and a reduction in nucleic acid damage in mouse brains 24 h after FLASH RT (10 Gy, 1 pulse of 1.8 µs) compared to CRT (10 Gy, 0.1 Gy/s). Notably, significant changes in lipid acyl chain conformations were observed in the asymmetric CH_2_/CH_3_ ratio, which reflects lipid chain length. In CRT, this ratio is decreased in some regions, indicating increased CH_3_ (methyl) and decreased CH_2_ (methylene) concentrations, which are changes linked to lipid peroxidation. In contrast, no such changes were detected in the FLASH RT and the unirradiated control groups, highlighting distinct lipid structural alterations between FLASH RT and CRT. The study emphasized that biochemical changes are observed after FLASH RT (vs. CRT) which not only involves nucleic acids, but also proteins and lipids, indicating a complex response. This further encourages the investigation into the effects of FLASH RT on lipid metabolism and peroxidation.

These studies underscore the importance of the cell membrane composition in the effects of FLASH RT, showing that it induces unique alterations in lipid metabolism, including lipid peroxidation and structural changes, with varying responses in normal and tumor tissues.

### 4.4. Limitations in Evaluating Lipid Peroxidation Following FLASH RT

While LA in the lipid vesicles represents the predominant PUFA in cell membranes, enabling investigation into the different reactions of the chain reaction, the lack of complexity, along with the absence of AO and a cellular context, makes drawing the parallel to biological membranes challenging. With no studies published regarding the effect of FLASH RT on ferroptosis, this research could broaden our understanding of the biological impact of FLASH RT.

While several studies have included variations in oxygen levels [15,103,109], it would be interesting to investigate FLASH RT on lipid peroxidation under hypoxic conditions for several reasons. First, almost all solid tumors are characterized by hypoxia [111], so studying this would provide insights into how the hypoxic tumors act upon FLASH RT, rather than focusing solely on the normoxic, healthy tissue, which often serves as proxy for understanding the FLASH effect. Second, FLASH RT consumes oxygen. While oxygen depletion alone is considered not responsible for the FLASH effect, a temporary induction of hypoxia would theoretically provide radioprotection to both healthy and tumor cells. Third, there is ongoing debate regarding the effect of hypoxia on lipid peroxidation and ferroptosis. While it is established that hypoxia upregulates ROS production [112,113], which in principle would result in more lipid peroxidation and ferroptosis to take place, other studies reveal that lipid peroxidation is not increased post FLASH RT. Instead, it remains consistently lower than with CRT, despite lower oxygen levels [15,103]. Mounting evidence indicates that certain hypoxia-related genes suppress ferroptosis, consistent with the observation that FLASH RT promotes oxygen consumption and reduces lipid peroxidation [114,115,116]. Further research is needed to explore the role of lipid peroxidation and ferroptosis in the effects of FLASH RT, particularly using biologically relevant models.

## 5. Mitochondrial Metabolism in Ionizing Radiation

**Figure 4:**
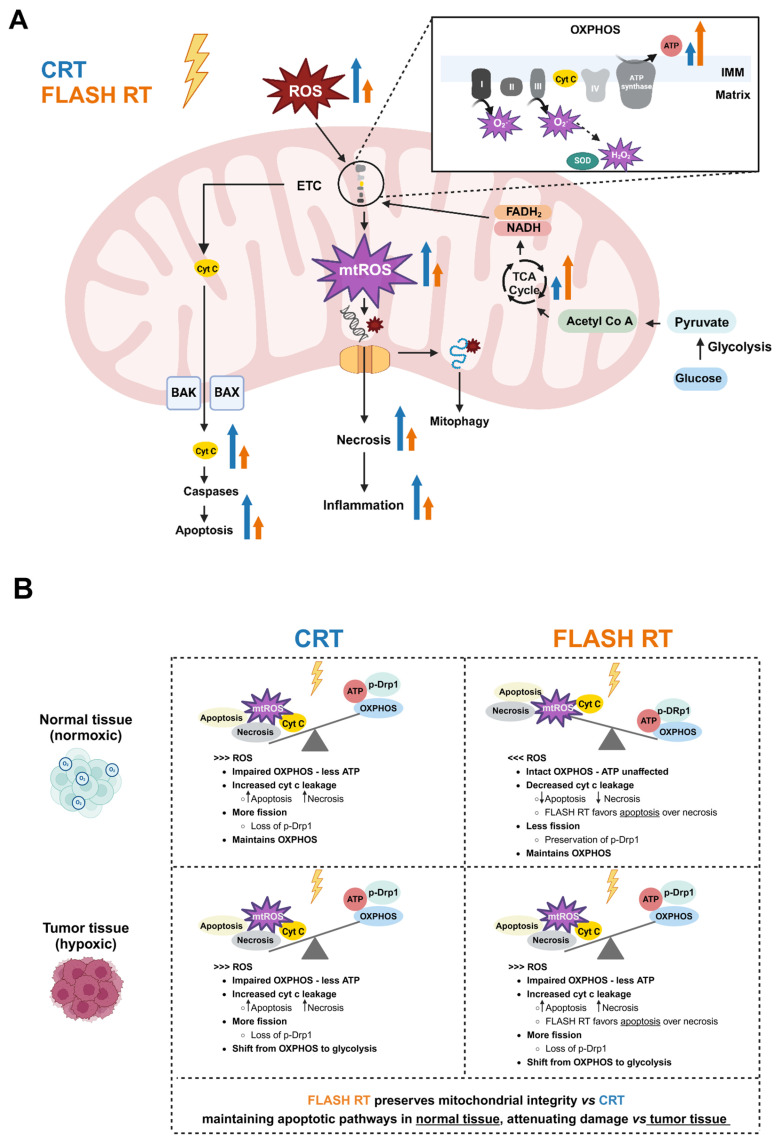
(**A**) Differential impact on mitochondrial metabolism following FLASH radiotherapy (FLASH RT) (orange) compared to conventional radiotherapy (CRT) (blue). Ionizing radiation (IR) generates reactive oxygen species (ROS) through water radiolysis and as byproducts of mitochondrial oxidative phosphorylation (OXPHOS). Mitochondrial ROS (mtROS) are predominantly formed at complex I, II, and III of the mitochondrial electron transport chain (ETC), converting superoxide (O_2_∙^−^) to hydrogen peroxide (H_2_O_2_) via superoxide dismutase (SOD). Cytochrome c is involved in mitochondrial respiration and ATP synthesis. Following glycolysis, the tricarboxylic acid (TCA) cycle generates NADH and FADH_2_, which fuel the ETC in the inner mitochondrial membrane (IMM). Excessive mtROS can damage mitochondrial DNA (mtDNA), disrupting the expression of mitochondrial respiration proteins. When oxidative damage surpasses repair mechanisms, mitochondria may be degraded through mitophagy. IR can activate pro-apoptotic proteins BAX and BAK, permeabilizing the outer membrane and causing cytochrome c leakage, leading to caspase activation and apoptosis. Mitochondrial disruption can release mtROS and mtDNA, potentially causing necrosis and inflammatory responses. (**B**) Side-by-side comparison of mitochondrial metabolism after FLASH RT versus CRT in normal and tumor tissue. (**Top left**): CRT elevates ROS levels in normal tissue, impairing OXPHOS and ATP production while elevating mtROS, damaging mitochondria. This results in cytochrome c release, apoptosis, and necrosis. CRT enhances mitochondrial fission via Drp1. Despite the CRT-induced changes, the metabolic profile remains OXPHOS-dependent. (**Top right**): FLASH RT results in less ROS formation in normal tissue, preserving OXPHOS and ATP production, maintaining mtROS levels, and sparing mitochondria. Cytochrome c leakage is reduced, favoring apoptosis over necrosis. FLASH RT preserves phosphorylation of Drp1 (pDrp1), preventing excessive fission and necrosis. The metabolic profile remains OXPHOS-dependent. (**Bottom left**): CRT elevates ROS levels in tumor tissue, impairing OXPHOS and ATP production while elevating mtROS, damaging mitochondria. This triggers cytochrome c release, apoptosis, and necrosis. CRT enhances mitochondrial fission via Drp1. The metabolic profile shifts from OXPHOS to glycolysis to adapt to hypoxia and IR. (**Bottom right**): FLASH RT elevates ROS levels in tumor tissue, impairing OXPHOS and ATP production, while elevating mtROS, damaging mitochondria. This results in cytochrome c release, favoring apoptosis over necrosis. CRT enhances mitochondrial fission via Drp1. The metabolic profile shifts from OXPHOS to glycolysis to adapt to hypoxia and IR.

While the role of mitochondria in (conventional) radiation responses has been extensively reviewed by others [117,118,119], a brief overview of the effects of IR on mitochondrial metabolism is necessary to draw parallels to FLASH RT in the remainder of this review (Figure 4A).

Radiation-induced cell death is mainly caused by oxidative damage to DNA. In irradiated tissues, ROS is generated through the radiolysis of water molecules and as byproducts of oxidative phosphorylation (OXPHOS) in the mitochondria. ROS are predominantly generated within the mitochondrial electron transport chain, especially at complexes I, II, and III [120].

The mitochondria dominate cellular metabolism, providing the energy necessary for essential functions and playing a pivotal role in signal transduction. While the mitochondria possess AO systems to manage ROS levels, excessive ROS caused by IR can exhaust the defenses, causing mitochondrial injury. A damaging cycle is created when IR-induced ROS impairs OXPHOS, which in turn amplifies ROS production. Damage to mitochondrial DNA, which is genetically much denser compared to that of the nucleus [121], alters the expression of proteins associated to mitochondrial respiration. When mitochondrial damage becomes excessive, these mitochondria are degraded through mitophagy [122]. Moreover, IR-induced oxidative damage permeabilizes the outer membrane through Bcl-2 proteins BAX and BAK, allowing cytochrome c release [123]. Cytochrome c is a mitochondrial protein that is involved in mitochondrial respiration via transporting electrons from complex III to IV and creating ATP synthase. Upon entering the cytosol, cytochrome c activates the caspase proteases which function as downstream effectors of apoptotic cell death [119]. Moreover, the release of mitochondrial ROS and DNA during the disruption of the outer membrane may also mediate inflammatory responses; however, this is beyond the scope of this review.

As such, mitochondria play an important role in cellular processes including energy production, apoptosis, and regulation of ROS, which are all processes that are dysregulated in cancer cells to sustain unlimited proliferation. A hallmark of cancer is the altered energy metabolism, where cancer cells shift from OXPHOS to glycolysis, also known as the Warburg effect. This process enables the cancer cells to produce the necessary building blocks via the glycolytic pathway, regardless of the availability of oxygen. While the phenomenon produces less ATP per glucose molecule in comparison to OXPHOS, it does provide a survival advantage to cancer cells in hypoxic conditions [124], which is typically found in the tumor microenvironment. The involvement of the Warburg effect also results in elevated AO production, supporting ROS homeostasis [125]. Furthermore, cancer cells commonly react to ionizing irradiation through temporarily shutting down mitochondrial activity [126].

More recently, targeting the mitochondrial metabolism to improve the therapeutic effects of IR has garnered interest. This includes shifting the metabolism of cancer cells from glycolysis to OXPHOS, promoting ROS production and, thus, the radioresponse [125,127,128]. Moreover, compounds inhibiting mitochondrial oxygen consumption have been found to elevate tumor oxygenation, improving their radiosensitivity [129,130,131].

In summary, understanding the interplay between IR, mitochondrial metabolism, and the resulting cellular responses is critical for elucidating the mechanism underlying FLASH RT, especially in how it may favor tumor cell death while sparing normal tissues.

### 5.1. Conceptual Framework of Mitochondrial Metabolism in FLASH RT

The demonstration of lower ROS in biologically relevant models, which can be explained by the current standing hypotheses, enhanced radical–radical recombination and transient oxygen depletion through enhanced oxygen consumption, makes the mitochondria a prime candidate for investigating the FLASH effect.

As described prior, cancer cells exhibit elevated endogenous ROS levels and enhanced mitochondrial functions to meet their metabolic needs and promote tumorigenesis. Due to their limited AO reserves, cancer cells may be prone to changes in mitochondria, leading to enhanced programmed cell death, while normal cells, with their greater AO capacity, are less susceptible to mitochondrial ROS changes, preserving cellular viability (Figure 4B).

Thus, it is crucial to explore whether mitochondrial-mediated oxygen consumption, as well as mitochondrial dynamics and function, differ between FLASH RT cells vs. CRT, and to determine whether these differences contribute to the protection of normal cells while still effectively targeting cancer cells.

### 5.2. In Vitro Studies of Mitochondrial Metabolism in FLASH RT

The following studies investigate the effect of FLASH RT on the mitochondria and their metabolism.

One of the first papers exploring the role of mitochondrial respiration following FLASH RT investigated murine embryonic fibroblasts under normoxic (21% O_2_) and hypoxic (mimicked by CoCl_2_) conditions [18]. FLASH RT (>10^9^ Gy/s ultra-fast laser generated particles) increased apoptosis and necrosis over time, but reductions in this effect were observed under hypoxia. This indicated that, similarly to CRT (0.05 Gy/s Co_60_ ү-radiation), hypoxic fibroblasts are more resistant to FLASH RT (compared to oxygenated fibroblasts). Using fibroblasts without cytochrome c, lower levels of late apoptosis and necrosis were demonstrated following FLASH RT, compared to their counterparts with functional cytochrome c, regardless of oxygen levels. Nonetheless, a comparison with CRT, alongside measurements of cytochrome c in the cytosol following FLASH RT, is necessary to validate that this is cytochrome c-driven apoptosis. Overall, this study suggests that hypoxia and mitochondrial dysfunction contribute to the radioresistance of mouse embryonic fibroblasts to FLASH RT, which underscores the potential significance of mitochondria in the FLASH effect.

Another study explored mitochondrial damage in proton FLASH-irradiated human lung fibroblasts (IMR90) under aerobic conditions (21% O_2_) [19]. In human lung fibroblasts, the mitochondria maintained their morphology and functionality after 15 Gy of FLASH RT (100 Gy/s), whereas CRT (0.33 Gy/s) caused structural abnormalities and impairment of functions (membrane potential and mitochondrial copy number). After FLASH RT (vs. CRT), the cells also consumed more oxygen, and ATP production was less impaired. In contrast, this mitochondrial preservation was not observed in lung cancer cells, indicating that FLASH RT could safeguard normal cells from mitochondrial damage, while still targeting cancer cells.

The study also associated the observed difference in mitochondrial impairment between FLASH RT and CRT to dephosphorylation of the dynamin-related protein (Drp1). Drp1 plays an important role in mitochondrial fission, a process associated to programmed cell death, where the mitochondria divide into two organelles, with the opposite event being fusion. While CRT increased Drp1 expression and colocalization with p53, these levels remained mostly unaltered after FLASH RT [19]. In concordance, fibroblasts primarily went into apoptosis after FLASH RT, whereas CRT led to necrosis. Subtle elevations were observed in expression of cytochrome c post FLASH RT, whereas it was lowered after CRT. The findings led to the hypothesis that FLASH RT prevents the dephosphorylation of Drp1 typically seen in CRT. The preservation of phosphorylated Drp1 inhibits its translocation to the mitochondria, thereby preventing mitochondrial damage and necrosis, while favoring apoptosis instead [19]. Consistent with the previous study [18], these findings suggest a connection between FLASH-induced apoptosis and mitochondrial respiration.

A recent non-peer reviewed preprint (bioRxiv: https://doi.org/10.1101/2024.04.10.588811) offers preliminary insights into the effects of FLASH RT on mitochondrial structure and dynamics. In contrast to the two earlier studies discussed, human breast cells (MCF-10A) exposed to FLASH electron irradiation (61 or 610 Gy/s) showed enhanced cytochrome c leakage but no morphological differences in the mitochondria (vs. CRT (0.36 Gy/s)). Additionally, breast carcinoma cells (MDA-MD-231) displayed less cytochrome c leakage and increased mitochondrial fission vs. normal cells.

In summary, these findings demonstrate how FLASH RT preserves mitochondrial function and integrity in normal cells, while tumors cells undergo more mitochondrial damage, contributing to the efficacy of FLASH RT.

### 5.3. In Vivo Studies of Mitochondrial Metabolism in FLASH RT

The differential responses of 20 Gy FLASH RT (125 Gy/s), compared to CRT (0.1 Gy/s), on esophageal tissue were investigated using histology and whole proteomic analysis [17]. The sparing effects of FLASH RT were validated, with 71% of esophageal tissue showing no signs of tissue damage, compared to only 14% in the CRT group. Additionally, the mitochondria in the FLASH-treated group showed less swelling, fewer autophagic vacuoles, and reduced infiltrating neutrophils compared to the CRT-treated group.

Proteomic cluster analysis identified variations in protein expression, particularly those related to mitochondrial function. The most pronounced differences between the two dose rates were observed in pathways associated to the tricarboxylic acid cycle, OXPHOS, and neutrophil-mediated immune responses. Following IR, it is well established that damaged mitochondria can trigger acute inflammatory responses, making this connection clear [123]. Protein expression involved in the 2 Tricarboxylic Acid (TCA) cycle, including for citrate synthase and pyruvate carboxylase, was reduced after CRT but appeared as only a partial reduction with FLASH RT. Similarly, OXPHOS-related proteins were also attenuated, including ubiquinol–cytochrome c reductase core protein 1 and ATP synthase Subunit b. Other important members of the mitochondria, including superoxide dismutase 1, showed a similar trend, in concordance with the previously described conceptual framework, which suggests that FLASH RT-induced ROS can be eliminated by the greater AO reserve in normal tissue, unlike in cancerous tissue with exhausted AO systems [39].

The impact of acute hypoxia on the sensitivity of an in vivo cancer model to FLASH RT was investigated [132]. Acute hypoxia in glioblastoma (U-87 MG) mouse xenografts did not reduce sensitivity to 20 Gy FLASH RT (100 Gy/s), whereas tumor clamping did make tumors more resistant to CRT [132]. The superiority of FLASH’s therapeutics effects towards clamped tumors was attributed to downregulation of certain gene sets including those related to translational functions. In addition, a metabolic shift from OXPHOS to glycolysis was observed 24 h post FLASH RT. Notably, both CRT and FLASH RT increased glycolysis gene expression, but only FLASH RT downregulated OXPHOS. The same study also observed that the combination of the glycolysis inhibitor trametinib enhanced the therapeutic effectiveness of FLASH RT in both clamped and non-clamped tumors, introducing the concept that targeting glycolysis could optimize FLASH RT in tumors. Nonetheless, this was not compared to tumors receiving CRT [132].

All together, these findings suggest the FLASH RT may mitigate tissue damage via reducing mitochondrial dysfunction and preserving cellular stability.

In short, FLASH RT offers more mitochondrial sparing compared to CRT, as evidenced by maintained mitochondrial function, reduced inflammation, and more cellular stability, while also proving effective under hypoxic conditions and when combined with glycolysis inhibition.

### 5.4. Challenges in Mitochondrial Metabolism Studies with FLASH RT

Certain challenges regarding the role of mitochondria and its metabolism in the underlying pathways of the FLASH effect need to be addressed.

Mitochondrial changes were observed in vitro 1 h post FLASH RT [19], while esophageal tissue was harvested for detecting mitochondrial alterations 10 days post irradiation [17]. Moreover, favoring apoptosis over the necrosis pathway after FLASH RT was transient for fibroblasts [19]. This highlights the need for a comprehensive time-course study of FLASH RT’s effects, extending for a long period of time.

While studies have documented reductions in extracellular oxygen consumption in normal tissue [19] and decreased OXPHOS gene expression in tumor tissue [132] after FLASH RT (vs. CRT), measuring kinetic changes in OXPHOS intracellularly could offer deeper insights in energy metabolism reprogramming. For instance, CRT-treated salivary glands showed an acute increase in intracellular oxygen consumption rate, which declined after 5 days, suggesting that targeting acute OXPHOS could potentially reduce ROS-inflicted damage and subsequent apoptosis [133]. Given that FLASH RT appears to mitigate mitochondrial impairment and subsequent apoptosis within a short time frame, it would be valuable to investigate whether this protective effect persists long-term or if any delayed effects exist.

While lung cancer cells (A549) were not guarded from mitochondrial impairment following FLASH RT under aerobic conditions, other studies have demonstrated a protective FLASH effect in other cancer cell lines [134]. Furthermore, hypoxia did not offer radioresistance in murine tumors with up to 20 Gy of electron FLASH RT (100 Gy/s), in contrast to CRT [132]. Interestingly, normal fibroblasts exhibited resistance to FLASH-IR under hypoxic conditions, further complicating the question of whether the FLASH effect is oxygen dependent. Oxygen levels are critical in regulating redox homeostasis, directly impacting mitochondrial respiration and endogenous ROS production. Future research should continue to incorporate cancer cell lines and hypoxia in experimental set-ups to fully explore the therapeutic potential of FLASH RT.

## 6. Potential Metabolic Avenues for Future Research

We have outlined the currently known impact of FLASH RT on various metabolic pathways, including ROS, iron, lipid, and mitochondrial metabolism. However, other metabolic areas targeted by IR warrant investigation to understand their interaction with FLASH RT. These include carbohydrate metabolism, protein metabolism, and DNA metabolism, as discussed below.

### 6.1. Glycolysis, Tricarboxylic Acid (TCA) Cycle, and Mitochondrial Respiration

Other key metabolic pathways, such as the TCA cycle and the pentose phosphate pathway (PPP), are interconnected with glycolysis and mitochondrial respiration and are integral to the carbon metabolism responsible for energy production [135,136]. To cope with the IR-induced ROS production and subsequent cellular stress, cancer cells often enhance glycolysis for ATP production, despite oxygen-rich conditions, while also using the TCA cycle and mitochondrial respiration for supplementary energy and biosynthetic precursors [137]. In contrast, normal cells rely on mitochondrial respiration and the TCA cycle for ATP production [138,139].

### 6.2. Pentose Phosphatase Pathway (PPP) and One-Carbon Metabolism

Following IR, PPP is activated and produces nicotinamide adenine dinucleotide phosphate (NADPH) and ribose-5-phosphate, which can mitigate oxidative stress and facilitate DNA repair [140]. The response of normal vs. tumor tissues to FLASH RT-induced DNA damage may differ, as discussed elsewhere [141,142]. The activation of DNA damage response pathways following IR, such as homologous recombination and non-homologous end joining, elevates the demand for nucleotides and amino acid synthesis. Amino acids such as glutamate and serine provide building blocks for nucleotides and glutathione to support cellular recovery and redox homeostasis [110,143]. Additionally, one-carbon metabolism is stimulated following IR, providing essential precursors and methyl groups necessary for nucleotide and amino acid synthesis, which aid cellular recovery.

### 6.3. FLASH RT in Metastatic Tumors and Abscopal Effect

The application of FLASH RT in metastatic tumors represents a particularly promising area of research. Metastatic tumors have heterogenous microenvironments, characterized by variations in metabolic activity and oxygenation. FLASH RT, with its ability to mitigate normal tissue toxicity while exploiting the limited AO capacity of tumor cells, holds the potential to improve therapeutic outcomes by decreasing off-target effects. The FAST-01 trial already investigated proton FLASH RT (≥40 Gy/s) in 10 patients with bone metastases [144]. This study shed light on the advantages of proton FLASH RT over electron FLASH RT, including greater penetration depth and superior dose uniformity, and demonstrated its clinical feasibility, supporting further exploration of this technique in metastatic settings. The FAST-02 trial in patients with thorax metastasis is currently ongoing.

Further research could also explore potential abscopal effects of FLASH RT, which may contribute to the development of combinatory treatments involving FLASH RT and immunotherapy. While these topics are beyond the scope of this review, we recommend consulting these reviews [145,146,147] for a more comprehensive discussion.

### 6.4. The Role of Hypoxia in FLASH RT

We have outlined the current understanding of FLASH RT on both normal and cancerous tissue, distinguishing between hypoxic and normoxic conditions where applicable. However, the specific role of hypoxia in FLASH RT warrants further investigation. Early theories suggested that the FLASH effect could be attributed to the transient hypoxic induction of normal tissue cells [39]. However, subsequent research has refuted the oxygen depletion hypothesis, leading to a more nuanced understanding of oxygen’s role in the FLASH effect [40,41,42,44]. In general, our review discusses how CRT elevates ROS in tumors but faces hypoxia-driven resistance, whereas FLASH RT generates higher ROS, exploiting the tumor’s limited AO capacity with minimal oxygen consumption. Furthermore, in silico models show that oxygen depletion at clinically relevant doses can be neglected in normal tissues [43,44]. Experimentally, FLASH RT retained antitumor efficacy under acute hypoxia [132]. These findings suggest that endogenous tissue oxygenation seems to play a prominent role in the effect of FLASH RT; although, the extent of this should be further investigated. For further insight, we refer to reviews that provide a comprehensive discussion of the complexities of hypoxia, including the role of HIF1α, in FLASH RT [29,30,147,148,149,150].

In summary, exploring the potential parallels between FLASH RT and the impact of CRT could provide valuable insights and implications for enhancing the therapeutic window.

## 7. Conclusions

FLASH RT represents a promising advancement in cancer treatment that effectively protects healthy tissues form radiation-induced toxicity without compromising tumor effectiveness, a phenomenon referred to as the FLASH effect [5,6,7,8,9]. Distinguished by its UHDRs, FLASH RT has the potential to significantly enhance the therapeutic window; although, the specific mechanisms behind the FLASH effect remain largely unknown. Here, we discussed the impact of FLASH RT on various metabolic pathways, which yield insights for further exploration of the processes involved in the FLASH effect and its clinical applications.

Studies have demonstrated that FLASH RT significantly influences ROS dynamics. By generating a higher number of ionization events in a shorter time frame than CDR, FLASH RT is proposed to promote radical–radical recombination, leading to lower ROS levels and reduced cellular damage. The greater defense systems in normal cells compared to cancer cells explain the targeting of tumors while sparing healthy tissues. Nonetheless, most preliminary studies on redox metabolism have been conducted in aqueous cell-free solutions, stressing the disparity between these findings and their relevance in a biological context.

Additionally, FLASH RT has been associated with a reduction in lipid peroxidation vs. CRT. The complex interplay of lipid hydroperoxides, Fe^2+^, and elements such as MDA creates a cascade that may lead to ferroptosis-mediated cell death. The heightened sensitivity of tumor cells to FLASH RT is possibly attributed to their higher levels of Fe^2+^ necessary for lipid peroxidation, which further underscores the biological differences between cancer and normal tissues. This metabolic area warrants further exploration, particularly because no studies have examined the impact of FLASH RT on ferroptosis, the Fe^2+^-mediated cell death that results from lipid peroxidation.

Mitochondria also plays a pivotal role in mediating the effects of FLASH RT. The studies indicate that FLASH RT tends to preserve mitochondrial morphology and function in normal cells while causing significant damage to cancer cells. The preservation of mitochondrial integrity is linked to the maintenance of apoptotic pathways, which could explain the attenuation of damage in healthy tissues. Nonetheless, varying observations in lipid peroxidation following FLASH RT could be clarified through introducing longitudinal studies that examine temporal dynamics of mitochondrial responses.

Currently, many different modalities are being used to achieve FLASH RT, including photons, electrons, protons, carbon ions, and laser-generated beams, and due to limited available studies, we have made no distinctions for the beam-types’ metabolic impact. Additionally, dosimetry, which could react differently in terms of response time and saturation effect, mean dose rate (MDR), and pulse structure, including dose per pulse (DPP), is often underreported. Given the potential influence of these modalities on the research findings, as extensively described elsewhere [151,152,153,154], we acknowledge that current interpretations should be approached with caution.

Future research should prioritize integrating various biological systems, including both normal tissue and cancer cell lines in addition to tissues, rather than limiting investigations to aqueous studies. Exploring the implications of hypoxia and examining the kinetic changes in OXPHOS, along with its involvement in downstream metabolic pathways, is essential for uncovering the therapeutic potential of FLASH RT.

In conclusion, FLASH RT represents a leap forward in RT, offering unique mechanisms that hold the potential for improved tumor control and reduced damage to normal tissues. As research continues to unravel the complexities of this novel treatment, it may pave the way for more effective and targeted cancer therapies, ultimately improving patient outcomes.
